# Modeling Internal Movement of Children Born in Hong Kong to Nonlocal Mothers

**DOI:** 10.3390/ijerph17155476

**Published:** 2020-07-29

**Authors:** Paul Yip, Mehdi Soleymani, Kam Pui Wat, Edward Pinkney, Kwok Fai Lam

**Affiliations:** 1 Department of Social Work & Social Administration, The University of Hong Kong, Hong Kong, China; 2 Department of Statistics, Auckland University, Auckland 1142, New Zealand; mehdi.soleymani@me.com; 3 Department of Statistics & Actuarial Science, The University of Hong Kong, Hong Kong, China; watkp@hku.hk (K.P.W.); hrntlkf@hku.hk (K.F.L.); 4 The Hong Kong Jockey Club Centre for Suicide Research & Prevention, The University of Hong Kong, Hong Kong, China; edwardpinkney@googlemail.com

**Keywords:** return rate, China, Markov chain, migration, Hong Kong

## Abstract

In Hong Kong, approximately 300,000 children were born to Mainland China couples in the period 1991–2012. According to Basic Law, the mini constitution of Hong Kong Special Administrative Region (SAR) government, these parents do not have residence rights, but their children do. As a result, most of these children have returned to Mainland China with their parents. An important consideration for policymakers is how many of these children (who are now adults in some cases) will return to Hong Kong for good, and when, as this will have a significant impact on social service provision, especially in the education sector, where it will be necessary to ensure there is capacity to meet the additional demand. Prior survey results conducted by the government suggested that more than 50% of these children would return to Hong Kong before age six. It is important to be able to provide a timely projection of the demand into the future. Here, we make use of the immigration records on the actual movement of these children and propose a Markov chain model to estimate their return rates in the future. Our results show that only about 25% of these children would return rather than 50% estimated by the survey. We also find that parents with better educational attainment levels are associated with lower return rates of their children. Timely and relevant social and public policies are needed to prepare for their return to minimize disruption to the local population and promote social harmony for the whole community.

## 1. Introduction

Hong Kong, a former British colony, has been a Special Administrative Region (SAR) of the People’s Republic of China since 1997. It is one of the most densely populated modern metropolises in the world with a population of 7.4 million in an area of 1000 square km, and only 7% of its land is for residential use. Hong Kong has also undergone a major demographic transition in the past few decades. It has both a persistently low fertility rate of 1.2 per woman [1,2] and also one of the highest life expectancies globally, with ages 84 and 88 for men and women, in 2018, respectively. The proportion of older adults (aged 65 or over) was 18.0% in 2018 and is expected to rise to 33.1% by 2064 [3,4]. There are serious concerns about an aging and shrinking workforce in the population. What is unique to Hong Kong is not aging itself but its pace and magnitude. For instance, it has taken France 100 years to double its older adults’ population from 7% to 14%, whereas a doubling in Hong Kong took only 25 years [2]. To help address this, improving fertility rates has been one of the strategic population goals of the Hong Kong SAR government [5,6]. However, the marriage rate has not increased, and the divorce rate has gone up; both of these have had a negative impact on fertility [7]. The total fertility rate still remains at a very low level of 1.1 per woman. 

Nevertheless, cross border activities between Mainland China and Hong Kong, since 1997, have increased substantially in terms of trade, education, tourism, and even marriages. Cross-border marriages of Hong Kong residents to Mainland China counterparts have accounted for over 30% of all registered marriages in Hong Kong since 2003 [3]. However, there are still restrictions on Mainland China spouses joining their partners in Hong Kong due to the large population size. A one-way permit system is in place which allows a daily quota of 150 Mainland China residents for family reunions. To date, over one million residents have migrated to Hong Kong in the past two decades, and they are mainly the spouses and children of Hong Kong residents [6]. 

Furthermore, according to Basic Law, the mini constitution of Hong Kong SAR, since 1997, any children born in Hong Kong to Mainland China parents, irrespective of their residence status, are automatically granted permanent residence in Hong Kong. These children are entitled to all social welfare and government services, including school education and healthcare [8]. It is very appealing to Mainland China couples to give birth in Hong Kong for several reasons. Firstly, due to the one-child policy in Mainland China, which was enacted in 1979, couples in urban region could only have one child; otherwise they would face severe punishments such as monetary fines and, sometimes, forced abortion. The one-child policy in Mainland China has recently been relaxed due to an acute aging problem. Secondly, residence rights in Hong Kong SAR are very attractive to mainlanders because the Hong Kong SAR passport has fewer international travel restrictions than the Mainland China passport. Hong Kong SAR passport enjoys a visa-free arrangement to 158 countries, as compared with only 58 countries for the Mainland China passport.

There have been more than 300,000 children born so far in Hong Kong to Mainland China parents, since 1997. Initially Hong Kong SAR government did not do anything to regulate the flow of Mainland China parents giving birth in Hong Kong as the government saw the arrangement as an opportunity to rejuvenate its population and slow down population aging. By 2012, however, the number of births from nonlocal mothers were nearly 40% of the total. The rapid pace and large magnitude of the increase in number of these births has left the whole Hong Kong community unprepared due to insufficient provision of school and health services. For example, local children are having to go to other districts to attend school, mothers are having to compete for hospital beds for delivery and milk powder for feeding babies. The excessive demand has also made all of these services more expensive, causing considerable discomfort in the local community and further straining the relationship between mainlanders and Hong Kongers. The recent social unrest in Hong Kong, since June 2019, could be moderately related to the insufficient handling of the Mainland China migrants and visitors to Hong Kong, with excessive numbers possibly worsening congestion in the community.

Most of the children under consideration return to Mainland China after birth as their parents have no right of residency in Hong Kong. However, because of parents’ preference for the free, high quality Hong Kong education system, there are many who consider moving back to the territory or cross the border daily for their children’s schooling [9]. These additional school age children have already posed significant challenges to our existing school system, especially to those schools in the districts close to the Mainland China border. There are also concerns about the well-being of these children, as some may be looked after by their extended relatives and appointed guardians. This separation could affect the bonds between parents and children, and lead to heightened risk of social adjustment problems, low self-esteem, and depression [10,11]. As such, it is concerning that there continues to be much uncertainty on the number of potential future returnees. 

Currently, the return rates are estimated based on a government survey collected when parents present to the Immigration Department to collect the birth certificate of their children in the first 42 days after birth, according to the law of birth registration. The survey results based on parents’ replies suggested that about 50% of these children return to Hong Kong before age six [3].

The Census and Statistics Department (C&SD) is the department which is responsible for the population forecast. All major infrastructure planning and development activities rely on its accurate population projection. However, the accuracy and validity of these estimates has become a major concern as the information on returning was collected at the time of application of birth certificates, and the return of children can be many years later and circumstances could have changed. There is an urgent need to monitor and predict return rates accurately for social services planning, especially in view of the rapid changes to the socioeconomic environment in Hong Kong and Mainland China in recent years. Oversupply and under-preparedness are equally negative, in any social welfare and school planning. Recently, continuous improvements in the provision of education services and quality of life in Mainland China could have diminished the intention of these mainland parents to bring their children to Hong Kong, and the hope of relying on these children to slow down the aging problem might not be justified.

In this paper, we use actual arrival and departure records of these children at Hong Kong’s borders and summarize the patterns of movement of children born in Hong Kong to nonlocal mothers. We use a Markov chain to model movement and predict return status [12,13]. We also examine the parents’ educational attainment profiles of children who have returned to Hong Kong and compare them with those who have not returned. We discuss some related public and social policies to prepare for the arrival of these children. 

## 2. Data

### 2.1. Types of Babies

Hong Kong SAR government categorizes the babies who were born in Hong Kong to Mainland Chinese women as Type A and Type B children, where Type A children are those whose fathers are Hong Kong residents and Type B children are those whose fathers are not Hong Kong residents. 

These children (whether Type A or Type B) are officially classified as Hong Kong permanent residents and have the right to gain access to the same resources as local children. Approximately 40% of all births in Hong Kong during the period 2004–2013 were made up of Type A (approximately 10%) and Type B (approximately 30%) children. The impact on the hospital delivery services of such an increased number of births, and the effect on the normal lives of Hong Kong residents, caused a considerable negative reaction in the community. Hong Kong SAR government amended the law, in 2013, so that no Mainland Chinese women can give birth in Hong Kong hospitals. Consequently, the number of births in Hong Kong hospitals by Mainland Chinese women has drastically declined from approximately 30,000 in 2012 to a few hundred since 2013. Nevertheless, there were 300,000 of these babies born during the period 1997–2012. The parents of Type A and Type B children usually bring their newborn babies back to Mainland China approximately one month after the birth date. In later years, these children return to Hong Kong for different purposes, such as leisure, medical treatment, schooling, and so on. 

The movement dataset considered in this paper was made available by the Immigration Department of Hong Kong SAR government and consists of the records of the movement in and out of Hong Kong customs of 305,945 Type A and Type B children, between 2004 and 2016. For each child, the dates of departure and arrival were obtained and the pattern of movement for each child was calculated by the order of their records. Since there was a chance that a child had more than one record on a specific day, we assumed that the arrival happened before the departure. The family background of each case was retrieved from a separate dataset and combined with their movement patterns. The information on the family backgrounds of the children was obtained from the information forms the parents completed upon the birth of their babies. The forms contained information such as occupation, education level, residence, and age of the father and mother. However, as the forms were self-administrated and it was not compulsory to complete them, some of the fields in the data could be missed. Consequently, in some of the list-wise statistical procedures, there were some variations in the total number of data items available for calculation purposes. We mainly looked at the most important variable, educational attainment level of the parents which was highly associated with their occupational status. 

The birth cohort, in the current study, was set for all the children born between January and December in a specific year. We calculated the noncumulative return rate for each birth cohort, which gave the percentage of returning children in a certain year. According to the definition given by the Census and Statistics Department (C&SD), a child is regarded as having “returned” to Hong Kong if they stay in Hong Kong for at least one month (31 nights, not necessarily consecutive) during a six-month period either before or after mid-year (i.e., 30 June of each year). For each child, we computed the number of nights they stayed in Hong Kong for each year. Children who had already lived in Hong Kong were classified as “returned”. 

### 2.2. Statistical Analysis

We proposed a Markov chain model [14,15,16] to predict the return rates. In this subsection, we briefly introduce the methodology and provide the results based on such a model. Gulshan, Mina and Hassain [17] used Markov chains to study the migration pattern in Bangladesh, and Zhang and Medoff-Cooper [18] used a Markov regression to fit a binary time series to time dependent data collected at the Hospital of University of Pennsylvania. They used the regression model to study the nutritive sucking and physiology status of premature infants. Muenz and Rubinstein [19] applied two logistic models to estimate the transition probabilities of a discrete Markov chain.

Let $Y_{n}\in \left\{0,1\right\},n=0,1,\ldots$ be the “returned” status of a child at age n, where 0 stands for “not returned” and 1 stands for “returned”. We assume that the status of a child at age n+1 only depends on their status at age n (i.e., probability of changing the status for a child only depends on their previous year’s status):

(1) $$\mathrm{Pr}(Y_{n+1}=y_{n+1}|Y_{0}=y_{0},\ldots ,Y_{n}=y_{n})=\mathrm{Pr}\left(Y_{n+1}=y_{n+1}|Y_{n}=y_{n}\right)$$

where $y_{j}\in \left\{0,1\right\},j=0,\ldots ,n+1$. The terms$\mathrm{Pr}(Y_{n+1}=0|Y_{n}=0)$, $\mathrm{Pr}(Y_{n+1}=0|Y_{n}=1)$, $\mathrm{Pr}(Y_{n+1}=1|Y_{n}=0)$, and $\mathrm{Pr}(Y_{n+1}=1|Y_{n}=1)$ indicate the probability that a child will not return at age n+1 given they do not return at age n, the probability that a child will not return at age n+1 given they return at age n, the probability that a child will return at age n+1 given they do not return at age n, and the probability that a child will return at age n+1 given they return at age n, respectively.

Under this assumption, we have a homogeneous two-state discrete Markov chain with the following transition matrix for a child:

(2) $$\begin{array}{cc} & \begin{array}{cc} Y_{t}=0 & Y_{t}=1 \end{array}\\ \begin{array}{c} Y_{t-1}=0\\ Y_{t-1}=1 \end{array} & \left(\begin{array}{cc} \theta_{1} & 1-\theta_{1}\\ 1-\theta_{2} & \theta_{2} \end{array}\right) \end{array}$$

where $\theta_{1}$ is the probability that a child will not return given they do not return in the previous year, and $\theta_{2}$ is the probability that a child will return given they return in the previous year. To simplify the model, we assume that both $\theta_{1}$ and $\theta_{2}$ depend on the birth cohort. Thus, the transition matrix has a form of

(3) $$\left(\begin{array}{cc} \theta_{1}^{j} & 1-\theta_{1}^{j}\\ 1-\theta_{2}^{j} & \theta_{2}^{j} \end{array}\right)$$

where j indicates the birth cohort. We estimate $\theta_{1}^{j}$ and $\theta_{2}^{j}$ on the basis of the return pattern of cases in the database, see [20]. This method has been increasingly used in labor economics and demographic research, especially in evaluating social policies [21,22,23].

## 3. Results

**Figure 1:**
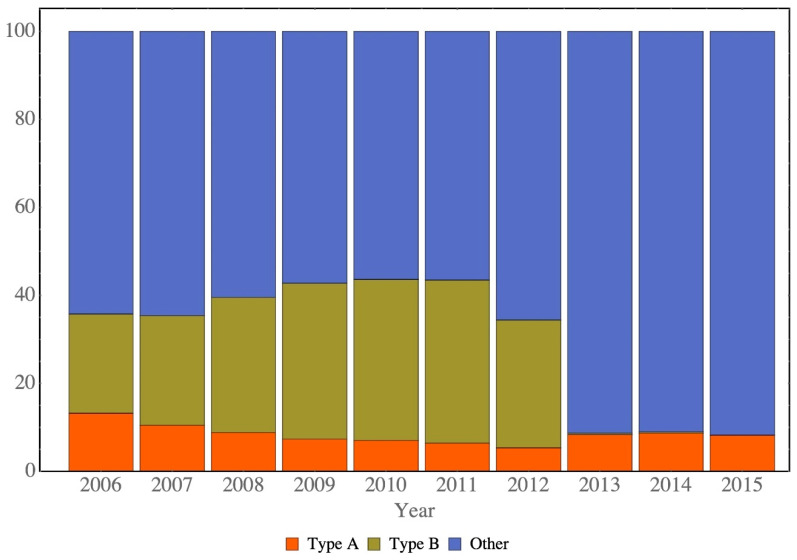
Distribution of Types A and B and other children from 2006 to 2015.

The distribution of the number of births was calculated on the basis of the birth information (Table 1). The term “total births” means the total number of live births in Hong Kong in a given year. In 2014, the number of Type A children decreased slowly from 2006 to 2012, and the number of Type B children increased from 2006 to 2011, followed by an abrupt decline in numbers since 2013. The decline in the number of Type B children was due to the introduction of the government’s policy of not allowing mainlanders to give birth in Hong Kong, in response to community outcry over Type B births overstretching maternal service in Hong Kong (Figure 1). 

**Figure 2:**
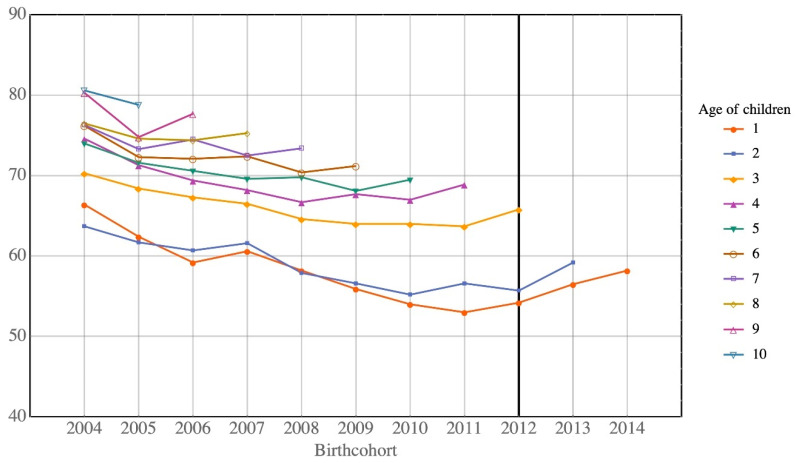
Return rates of Type A children of all ages by birth cohort.

**Figure 3:**
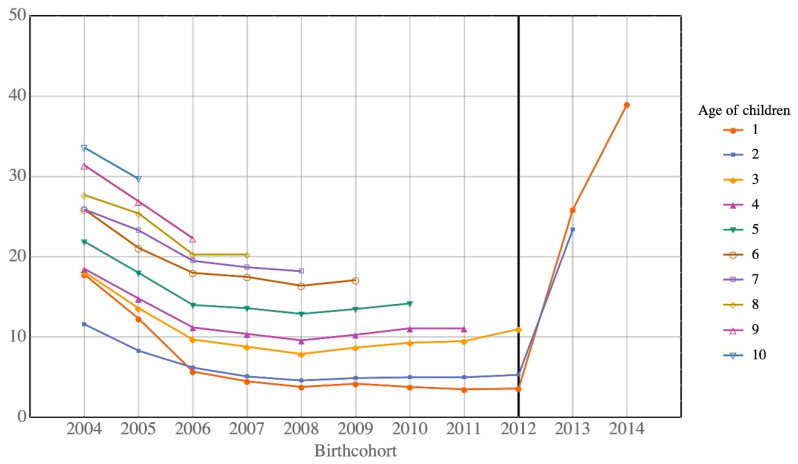
Return rates of Type B children of all ages by birth cohort.

Table 2 shows that the percentages of children returning to Hong Kong decrease for almost all ages. Figure 2 and Figure 3 show this trend for Type A and Type B children, respectively. The decreasing return rate for Type B children changes for children born since 2012, as depicted in Figure 3. The sharp increase in return rate is due to the small number of Type B children born after 2012 (211 births in 2013 and 165 births in 2014), following the implementation of the government policy of not allowing mainlanders to give birth in Hong Kong. The impact of the children born after 2012 is very limited due to its small number. Our discussion focuses mainly on the babies born in 2012 or before.

### 3.1. Return Rates by Parents’ Education Level

Table 3 shows the return rates, in 2016, based on the mothers’ education level by birth cohort. For all education levels of mothers, Type A children usually had a higher return rate than Type B children. For both Type A and Type B children, there was a higher probability of mothers with a lower education level bringing their children back to Hong Kong, except in 2013, when there were very few Type B children. It should be noted that the return rates of children whose mothers received no schooling or only kindergarten education varied enormously across years, as the number of these mothers was very small (Table 3).

Table 4 shows the return rates in 2016 based on the fathers’ education level by birth cohort. The pattern of return rates based on the fathers’ education level is very similar to that of the mothers’ education level, in which lower return rates are usually found in children whose fathers received a tertiary (degree) education.

### 3.2. Estimating Future Return Rates 2016–2030

**Figure 4:**
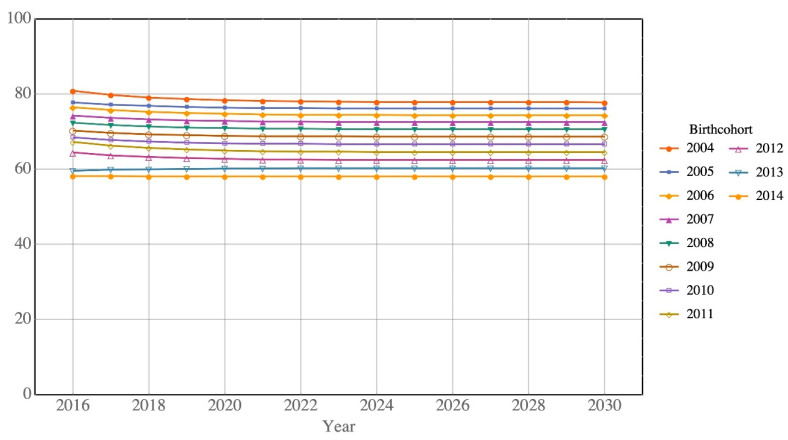
Predicted return rates of Type A children by birth cohort.

**Figure 5:**
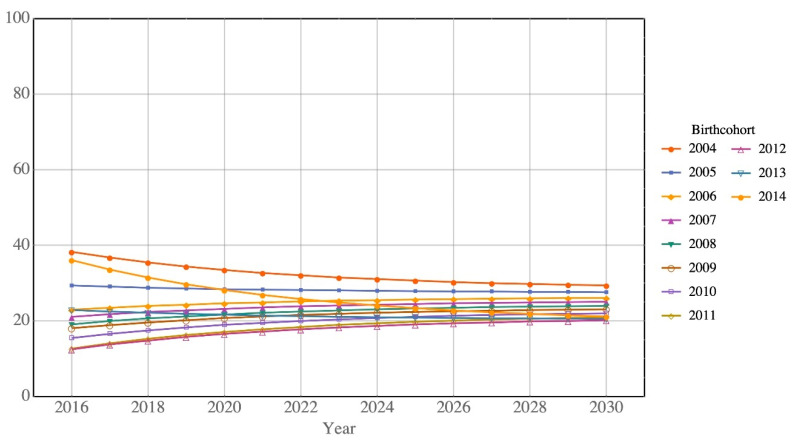
Predicted return rates of Type B children by birth cohort.

We use the estimated transition matrix to predict the return rate of children. Figure 4 and Figure 5 depict the predicted return rates by birth cohort. For Type A children, Figure 4 clearly shows that the return rate decreased with more recent birth cohorts, i.e., 77.8% (born in 2004) to 58.1% (born in 2014) of Type A children will return to Hong Kong in 2030. For Type B children, the predicted return rates are much lower than those of Type A for all birth cohorts, ranging from 20.2% (born in 2012) to 29.4% (born in 2004) in 2030 (Figure 5). 

As shown by the noncumulative return rates, it is predicted that only 77.8% of Type A children born in 2004 will return to Hong Kong by age 21 (i.e., by 2025). The corresponding proportion for Type B children is 30.7%, which is similar to the official C&SD estimation. However, the predicted return rate generally decreases with years of increasing birth cohort. For example, for the birth cohort 2012, the predicted return rates drop to 62.5% for Type A and 20.5% for Type B children by the age of 21 (i.e., year 2033). Amongst the 65,753 Type A and 195,042 Type B children born between the birth cohorts 2004 and 2014, under assumptions used by C&SD, there will be around 124,000 children returning to Hong Kong by age 21. Under the predicted return rates in this study, the corresponding number is only about 89,000. The estimated difference of 35,000 fewer children and young people returning is very substantial.

## 4. Discussion

We examined the historical Hong Kong return rates of Type A and Type B children and made predictions for future rates, which are crucial for social service provision. The number of returnees and also their return times are important considerations. The proposed Markov chain model has provided a framework to monitor the movement of these children. The findings estimate that, in 2016, about 71% of Type A and 16% of Type B children born on or after 1 July 2004 returned to Hong Kong. The parents of Type B children have better educational qualifications (on average) than those of Type A children, however, the better qualifications of the parents of Type B children are associated with a lower rate of return, i.e., it is less likely that their children will return to Hong Kong. If the government’s objective is to improve our human capital by recruiting Type B children and their families to Hong Kong, unfortunately, the evidence indicates that this is not currently proving to be successful. 

A comparison, with the estimates of the C&SD in Hong Kong Population Projections 2015–2064 [3] which provides 50-year projections based on previous information, indicates that our study predicts that there will be fewer than expected Type A and Type B children returning to and settling in Hong Kong. The official estimates, which were based on a survey collected at the time of birth of these children about the aspiration to be returned to Hong Kong, were outdated. Our proposed estimate used the actual return information based on the immigration data which gave much more reliable and updated information. The official estimate should be amended to more accurately examine the long-lasting impact that the number of returnees will have on population estimates in the community. Estimates using the most up-to-date information from this study should be taken into account in future population projections of Hong Kong.

In the period of increasing numbers of Type B babies born in Hong Kong, the government did not do much to improve or upgrade the infrastructure to mitigate the increased pressure on public facilities. It should have been a good opportunity to broaden the region’s economic activities, because increased migration usually supports economic growth. However, it is important to regulate its flow such that the community as a whole can absorb this scale of influx readily. The increase in the number of births by Mainland China parents in Hong Kong was so great, in such a short period of time, that the whole community was unprepared for the increase. These births from nonlocal parents could be a good replacement, because Hong Kong is very much in a low fertility trap with an average of only 1.1 children per woman and the chance of a fertility rebound by local women is very slim. The low fertility rate among Hong Kong women is not likely to improve in the near future, especially as aspirations of family formation and childbearing among the younger generation are not high [24,25]. Many countries have adopted replacement migration to attract young talented people to replenish a dwindling workforce. However, the inflow of these children from Mainland China needs to be monitored carefully and the sentiment of the local population needs to be carefully considered [26]. There have been many myths about the financial burden caused by Mainland China migrants. However, findings suggest that these Type B children and their families can offer new sources of labor to sustain Hong Kong’s development if integration can be managed properly [27]. The government should also explore how best to support the highly educated families of these children to migrate to Hong Kong with their children.

Limitations: The information collected by the Immigration Department is still quite limited, apart from some basic sociodemographic variables. It includes ages of the parents and educational and occupation information only. The variable of education is a good proxy for the financial status of parents in Mainland China. The financial status of parents is one of the more important factors to determine whether or not they aspire to send their children to Hong Kong. If the parents are of low educational attainment level, apparently, they will do better if they send their children to Hong Kong to receive all the free educational and medical benefits. However, for those with high educational attainment level, parents have more resources and opportunities beyond sending their children to Hong Kong. It seems that those who are well off would be less likely to send their children to Hong Kong before age six. They can afford to pay for nonlocal tuition fees (as their children do not have the hukou in the Mainland China) and they do not want to leave their children in Hong Kong with relatives. 

## 5. Conclusions

The movement data of Type A and Type B children captured by the Immigration Department provides very useful information about the population flow of Hong Kong’s population. A continuous monitoring and surveillance exercise should be in place to assess changes of inflow and outflow of the population. Some registry and monitoring, especially for Type B children, should be considered to track their movements in the future, and therefore to improve service planning.

## Figures and Tables

**Table 1:** Breakdown of numbers of various types of children who returned to Hong Kong in different years.

Year	Type A		Type B		Other		Total
2006	9732	(10.59%)	16,537	(18.00%)	65,626	(71.41%)	91,895
2007	8067	(8.22%)	19,198	(19.56%)	70,875	(72.22%)	98,140
2008	7510	(6.69%)	25,896	(23.07%)	78,822	(70.23%)	112,228
2009	6404	(5.38%)	30,597	(25.69%)	82,095	(68.93%)	119,096
2010	6611	(5.11%)	34,088	(26.37%)	88,584	(68.52%)	129,283
2011	6426	(4.63%)	37,023	(26.65%)	95,451	(68.72%)	138,900
2012	5073	(4.09%)	27,540	(22.18%)	91,558	(73.74%)	124,171
2013	5025	(8.06%)	211	(0.34%)	57,084	(91.60%)	62,320
2014	5777	(8.46%)	165	(0.24%)	62,305	(91.29%)	68,247
2015	5191	(7.97%)	57	(0.09%)	59,878	(91.94%)	65,126

**Table 2:** Percentages of children who returned to Hong Kong in different years.

Birth	Child	Year of Movement										
Year	Type	2005	2006	2007	2008	2009	2010	2011	2012	2013	2014	2015
2004	A	66.4	63.7	70.3	74.6	74.0	76.2	76.3	76.5	80.3	80.6	82.6
	B	17.8	11.6	18.1	18.5	21.9	25.9	25.9	27.7	31.4	33.6	40.0
2005	A		62.4	61.7	68.4	71.3	71.6	72.3	73.3	74.6	74.8	78.8
	B		12.3	8.3	13.6	14.8	18.0	21.1	23.3	25.4	26.9	29.7
2006	A			59.2	60.7	67.3	69.4	70.6	72.1	74.5	74.4	77.7
	B			5.7	6.2	9.7	11.2	14.0	18.0	19.5	20.3	22.3
2007	A				60.0	61.6	66.5	68.2	69.6	72.4	72.5	75.3
	B				4.5	5.1	8.8	10.4	13.6	17.5	18.7	20.3
2008	A					58.2	57.9	64.6	66.7	69.8	70.4	73.4
	B					3.8	4.6	7.9	9.6	12.9	16.4	18.2
2009	A						55.9	56.6	64.0	67.7	68.1	71.2
	B						4.2	4.9	8.7	10.3	13.5	17.1
2010	A							54.0	55.2	64.0	67.0	69.5
	B							3.8	5.0	9.3	11.1	14.2
2011	A								53.0	56.6	63.7	68.9
	B								3.5	5.0	9.5	11.1
2012	A									54.2	55.7	65.8
	B									3.6	5.3	11.0
2013	A										56.5	59.2
	B										25.9	23.4
2014	A											58.2
	B											39.0

**Table 3:** Return rates by mothers’ education level per birth cohort in 2016.

Birth	Child	Mothers’ Education Level					
Year	Type	0	1	2	3	4	X
2004	A	100.0	77.6	77.7	68.8	73.5	72.2
	B	50.0	41.0	39.5	45.5	38.9	33.3
2005	A	100.0	77.8	75.6	74.5	63.6	25.0
	B	66.7	31.1	30.4	25.0	22.6	50.0
2006	A	94.7	76.1	74.4	66.5	57.7	85.7
	B	26.4	21.9	22.1	18.5	18.1	50.0
2007	A	72.7	74.8	72.3	67.6	55.0	70.4
	B	18.9	21.4	20.1	19.9	17.4	19.8
2008	A	68.2	74.0	71.3	63.4	56.8	72.0
	B	13.6	19.3	19.5	15.2	15.1	16.2
2009	A	84.6	74.3	70.0	61.0	55.1	66.4
	B	17.9	20.7	18.7	14.9	14.5	18.5
2010	A	72.7	73.5	68.3	63.1	49.2	68.0
	B	16.0	18.7	16.4	13.9	11.6	18.9
2011	A	60.0	71.7	68.3	61.0	52.5	65.0
	B	13.9	13.7	12.9	10.6	11.3	11.3
2012	A	100.0	68.0	64.9	60.5	54.8	64.3
	B	20.0	14.6	11.9	11.2	10.7	11.7
2013	A	40.0	58.5	57.0	55.0	44.2	52.4
	B	0.0	60.0	29.3	19.2	12.5	19.5
2014	A	85.7	58.2	52.2	52.5	41.2	48.7
	B	0.0	0.0	22.4	12.0	37.5	15.6

Note: 0, no schooling/kindergarten; 1, primary; 2, secondary/matriculation; 3, tertiary (non-degree); 4, tertiary (degree); X, unknown.

**Table 4:** Return rates by fathers’ education level per birth cohort in 2016.

Birth	Child	Fathers’ Education Level					
Year	Type	0	1	2	3	4	X
2004	A	88.9	77.3	78.0	71.7	72.3	73.7
	B	0.0	41.4	39.4	34.4	29.2	47.7
2005	A	100.0	76.0	77.2	64.3	59.0	37.5
	B	60.0	32.3	29.3	24.5	28.3	43.1
2006	A	84.6	76.6	75.1	65.0	62.1	63.6
	B	18.8	20.9	21.3	20.9	18.0	39.0
2007	A	73.1	76.0	72.8	63.2	62.2	68.7
	B	12.5	21.1	19.6	18.4	17.3	30.4
2008	A	90.9	73.8	72.4	64.2	57.8	70.9
	B	20.6	18.2	18.5	15.9	14.9	25.0
2009	A	61.5	74.2	70.6	64.2	56.8	69.5
	B	21.6	19.5	18.1	14.8	14.5	13.3
2010	A	75.0	70.0	70.1	63.4	51.7	67.1
	B	27.8	20.0	16.0	13.4	11.5	18.3
2011	A	69.2	71.0	68.9	64.1	53.8	65.2
	B	10.3	17.3	12.3	10.4	10.4	11.5
2012	A	33.3	71.8	66.0	63.0	54.2	64.6
	B	8.3	13.0	11.3	10.5	10.5	11.1
2013	A	66.7	57.1	58.1	52.9	45.5	52.5
	B	0.0	50.0	26.3	26.3	19.5	21.6
2014	A	100.0	47.8	53.5	49.4	43.3	49.0
	B	0.0	0.0	21.6	13.6	35.7	13.8

Note: 0, no schooling/kindergarten; 1, primary; 2, secondary/matriculation; 3, tertiary (non-degree); 4, tertiary (degree); X, unknown.
